# The relationship between nomophobia and latent classes of personality

**DOI:** 10.1002/pchj.758

**Published:** 2024-05-01

**Authors:** Shunxin Ji, Suwei Xu, Zhao Zhou, Ye Zhu, Tour Liu

**Affiliations:** ^1^ Faculty of Psychology Tianjin Normal University Tianjin China; ^2^ Key Research Base of Humanities and Social Sciences of the Ministry of Education, Academy of Psychology and Behavior Tianjin Normal University Tianjin China; ^3^ Tianjin Social Science Laboratory of Students' Mental Development and Learning Tianjin China

**Keywords:** big five personality, latent class analysis, mobile phone use, nomophobia

## Abstract

The phenomenon of nomophobia, defined as the anxiety experienced when a person is without their mobile phone or is unable to use it, has been identified as having serious negative effects on individuals, particularly students. Previous research has explored the relationship between personality traits and nomophobia, but the findings have been inconclusive. The main objective of this study was to classify personality types through latent class analysis and explore the relationship between these personality types and nomophobia. The Chinese version of the Nomophobia Scale and the Chinese brief version of the Big Five Personality Inventory were used in this study to survey 1906 Chinese college students. The results indicated that (1) a four‐class model provided the best fit and categorized the personality traits as the overcontrolled class, resilient class, moderate class, and vulnerable class; (2) significant differences were observed between the four personality types and nomophobia, with overcontrolled and resilient personality types consistently scoring significantly lower than moderate and vulnerable personality types. Our finding highlights the key feature of the study.

## INTRODUCTION

According to the 52nd Statistics Report on China's Internet Development, the number of mobile phone users in China had reached 1.076 billion by June 2023 (China Internet Network Information Center, 2023). Smartphones have become one of the major devices used by people to obtain and transmit information (Bartwal & Nath, 2020). The rapid development of technology has led to continuous advancements in mobile phone functions, offering increased convenience for individuals. However, this convenience has also resulted in excessive dependence on mobile phones (Harris et al., 2020). Consequently, this excessive reliance has given rise to a phenomenon termed nomophobia (King et al., 2013).

Nomophobia has been the subject of widespread debate in recent years (Rodríguez‐García et al., 2020). Anshari et al. (2019) considered that nomophobia was a state of anxiety or unease caused by the inability to use a smartphone or by disconnection from the Internet. King et al. (2014) argued that nomophobia stems from an excessive dependence on virtual communication devices. Reduced mobile phone use can result in feelings of isolation, anxiety, low self‐esteem, and insecurity (King et al., 2013). Alternatively, Ren et al. (2023) argued that nomophobia consisted of both problematic mobile phone use and functional mobile phone use. Owing to the state of anxiety or problems caused by nomophobia, there has been a growing interest in studying it. Research have showed that nomophobia has a significantly negative impact on attention, cognition, and academic performance for students (Berdida & Grande, 2023; Gutiérrez‐Puertas et al., 2020; Schwaiger & Tahir, 2022). For example, a study by Berdida and Grande (2023) found that nursing students who possessed higher levels of nomophobia had less learning motivation and attention in the classroom, ultimately leading to a decline in their academic performance. Moreover, prolonged nomophobia can lead to anxiety and emotional instability, as well as to related psychological disorders such as depression, thereby affecting an individual's overall health (Darvishi et al., 2019; Farchakh et al., 2021; Liu et al., 2023). Hence, nomophobia has serious implications in individuals’ daily lives, and we continue to explore what factors influence its development.

Nomophobia, defined as problematic mobile phone use, has been extensively studied in relation to its antecedent variables. Previous research identified two main categories of variables that influence the emergence and development of nomophobia. The first category comprises demographic variables, such as gender, age, and education level etc. (Gezgin et al., 2018; Qutishat et al., 2020). For instance, Notara et al. (2021) discovered that young people were more susceptible to experiencing nomophobia than older people, while Arpaci (2022) found that women scored significantly higher than men in terms of nomophobia. The second category comprises psychosocial risk factors, including emotional dysregulation, solitude, and social support etc. (Pettorruso et al., 2020; Sahu et al., 2019). Previous studies have shown that emotional dysregulation and a lack of social support can increase the risk of addictive behaviors, including the problematic use of mobile phones (Gioia et al., 2021; Liese et al., 2020). Additionally, personality traits, which are stable psychological characteristics, play a significant role in influencing mobile phone use.

Brand et al. (2016) proposed the Interaction of Personality‐Affect‐Cognition‐Execution Model (I‐PACE), which suggests that personality traits act as predisposing factors that influence personal behavior towards mobile phone use. Dalbudak et al. (2020) further argued that different types of mobile phone use may be associated with specific personality traits. Therefore, different personality has varying effects on nomophobia (Li et al., 2023). Researchers have found that neuroticism and openness positively predict nomophobia (Liu et al., 2020; Sun et al., 2022; Zhang et al., 2020). Similarly, García‐Masip et al. (2023) demonstrated that extroversion was significantly positively related to nomophobia. On the other hand, Argumosa‐Villar et al. (2017) discovered that conscientiousness was a negative predictor of nomophobia. These findings highlight the significant role of personality in predicting nomophobia. It is clear that researchers often predict nomophobia from a single perspective of personality dimensions. This approach only reveals the relationship between the independent dimension and nomophobia (e.g., the positive prediction of nomophobia from neuroticism). However, it is worth considering whether we can examine the overall relationship between personality traits and nomophobia.

In studies exploring the effects of personality on mobile phone use, researchers primarily utilize variable‐centered approaches, which focus on the influence of separate personality traits on mobile phone use variables (Beierle et al., 2020; Marengo et al., 2020). It is particularly suitable for studying groups with similar characteristics, as it only considers issues that are common among the participants (Zeng et al., 2021). However, the person‐centered approach views personality as a whole and categorizes subjects into groups based on their response patterns, with each group exhibiting distinct personality features (Fisher & Robie, 2019). The person‐centered approach addresses the limitations of the variable‐centered approach by prioritizing the individual characteristics of subjects before examining their shared traits. Latent class analysis (LCA), as the person‐centered approach to identifying subgroups of people (Hofmans et al., 2020), is a valuable tool in this study. Researchers often utilize LCA to examine how various dimensions of a single variable interact within individuals and to identify heterogeneity among individuals. Lu et al. (2022) employed LCA to classify solitude behaviors into six distinct groups and to investigate the association between these different solitude groups and nomophobia. Hutchesson et al. (2021) employed LCA to identify various health behaviors and their correlations with psychological distress. Building upon this, in our study, we used LCA to categorize personality types and examine the variations in nomophobia across these different types.

The prevalence of nomophobia and its impact on the physical and mental health of individuals has been extensively documented in previous literature. Previous studies have also identified the effect of several variables on nomophobia. However, there is still a lack of clarity regarding the influence of personality on nomophobia, specifically because the focus only has been on the influence of different five personality dimensions on nomophobia, without taking into account their common effects. Therefore, based on the above, the issues to be addressed in this study are as follows: (1) identifying the personality types of Chinese students, and (2) investigating the differences in nomophobia among these personality types.

## METHOD

### Participants

A total of 1906 Chinese university students were recruited through an online questionnaire, with all participants providing complete responses. The majority of participants were aged between 17 and 24 years old (*M* = 17.65, *SD* = 5.38), and included 639 (33.5%) males and 1267 (66.5%) females. All participants provided written consent prior to their participation, and the study was approved by the Ethics Committee of Tianjin Normal University (XL2020‐08).

### Measures

#### Chinese Big Five Personality Inventory brief version

We used the Chinese brief version of the Big Five Personality Inventory (CBF‐PI‐B) revised by Wang et al. (2011). This version comprises five dimensions: Extroversion, Agreeableness, Conscientiousness, Neuroticism, and Openness. It consists of a total of 40 items, with eight questions for each dimension. A six‐point Likert‐type scale was used, ranging from 1 = *very inconsistent* to 6 = *very consistent*. The original article reported good reliability of the scale, with Cronbach α values of .80, .76, .81, .81, and .78 for the respective dimensions (Wang et al., 2011). In this study, the Cronbach *α* values of the five dimensions were .77, .78, .82, .86, and .87, respectively (where the order of the subscales was Extroversion, Agreeableness, Conscientiousness, Neuroticism, and Openness).

#### Nomophobia Scale

Yildirim and Correia (2015) initially developed the Nomophobia Questionnaire (NMP‐Q). In our study, we utilized the Chinese version of the Nomophobia Scale, which was revised by Ren et al. (2020) and consists of 16 items. The scale assesses four main reasons for experiencing fear without a mobile phone: (1) inability to communicate with anyone, (2) loss of contact, (3) inconvenience, and (4) inability to use. Therefore, the four dimensions of the nomophobia scale are labeled as “fear of being unable to obtain information”, “fear of losing convenience”, “fear of losing contact”, and “fear of losing Internet connection” (Moreno‐Guerrero et al., 2020). Participants rated the scale items on a 7‐point Likert‐type scale, ranging from 1 = *strongly disagree* to 7 = *strongly agree*. The original article reported good scale reliability, with Cronbach *α* values of .79, .82, .89, and .90 for each dimension (Ren et al., 2020). Additionally, in our study, the Nomophobia Scale demonstrated reliable measurement, with Cronbach α values of .79, .84, .89, and .91 for the respective dimensions.

### Data analysis

LCA is based on the principle that participants are grouped into classes by using their different response patterns on the categorical manifest variables as a reference standard (Wen et al., 2023). The latent class probability and the conditional probability, as two important parameters, are included in this analysis method. The latent class probability $[P\left(c_{i}=k\right)$] corresponds to the percentage of the total participants in a class (e.g., the latent class probability in C1 [class 1] is 70% if when 1000 subjects are divided into three categories and the number of people in C1 is 700). The conditional probability $[P\left(y_{i}=b_{i}ǀc_{i}=k\right)$] corresponds to the probability of an item ($b_{i}$) in the manifest variable (i) for an individual of class *k* (e.g., if a manifest variable has three items, the conditional probability of C1 is calculated as the proportion of individuals within C1 who choose between the three items of the manifest variable). The probability of participants at a certain answer level  is determined by summing of the product of the latent class probability and the conditional probability (Lu et al., 2022; Wang & Bi, 2020). The formula is as follows:

$$P\left(y_{i}=b_{i}\right)=\sum_{k=i}^{K}P\left(c_{i}=k\right)P\left(y_{i}=b_{i}ǀc_{i}=k\right).$$

In this study, participants' big five personality traits were used as categorical manifest variables to construct a meaningful and optimal number of latent class models using Mplus 8.0 (Muthén & Muthén, 2017).

Additionally, the effect of the latent class of college students' personality on nomophobia was further explored. After classifying personality, a one‐way analysis of variance (ANOVA) was performed on the divided classes and nomophobia scores using SPSS 19.0 (International Business Machines Corporation, New York, USA). However, relying solely on a one‐way ANOVA may introduce some error due to the uncertainty of the classes identified through the latent class model (Asparouhov & Muthen, 2014). To address this, the Bolck‐Croon‐Hagenaars (BCH) method was employed to validate the accuracy of the one‐way ANOVA findings.

## RESULTS

### Correlation analysis between personality and nomophobia

To examine the relationship between personality traits and nomophobia, a correlation analysis was conducted (see Table 1). The findings revealed a significant positive correlation between neuroticism and nomophobia variables (*rs*=0.34~0.48, *ps*<.01), including the total scores of nomophobia scale and four sub‐dimensions (information, convenience, contact and Internet connection). Additionally, Openness, Conscientiousness, Extroversion and Agreeableness were all significantly positively correlated with the “fear of losing contact”. Conversely, Conscientiousness exhibited a significant negative correlation with the total score and other dimensions of nomophobia, except for the “fear of losing contact”. Furthermore, extroversion showed a significant negative correlation with the “fear of losing convenience”, while agreeableness demonstrated a negative correlation with the “fear of losing Internet connection” (*r* = −0.06).

**TABLE 1:** Correlations between variables of personality and nomophobia.

Variables	M ± SD	1	2	3	4	5	6	7	8	9
1. Neuroticism	24.62 ± 7.94	1								
2. Openness	33.12 ± 7.22	−.071**	1							
3.Conscientiousness	34.01 ± 6.78	−.227**	.385**	1						
4. Extraversion	30.54 ± 6.37	−.247**	.467**	.320**	1					
5. Agreeableness	36.08 ± 6.10	−.091**	.397**	.463**	.284**	1				
6.Nomophobia	64.69 ± 20.38	.476**	.034	−.067**	−.006	.015	1			
7. Information	15.49 ± 5.33	.381**	.026	−.061**	−.004	−.035	.795**	1		
8. Convenience	15.79 ± 6.35	.477**	.006	−.106**	−.059 **	−.011	.877**	.647**	1	
9. Contact	18.63 ± 6.09	.340**	.082**	.059 *	.062**	.150**	.817**	.504**	.599**	1
10. Internet Connection	14.77 ± 6.44	.399**	.003	−.112**	−.016	−.055*	.870**	.575**	.688**	.634**

1 ** *

### LCA of personality

In this study, LCA was used to explore the latent class of personality traits. In order to find the best‐fitting model, we compared various statistics that assess model fit, including log likelihood, information criteria (Akaike information criterion [AIC], Bayesian information criterion [BIC], and sample size‐adjusted BIC [aBIC]), entropy, and likelihood ratio tests (bootstrapped likelihood ratio test [BLRT], Lo–Mendell–Rubin [LMR]). Smaller values for information indicators indicate a better model fit. An entropy value of 0.8 indicates that the model correctly classified 90% of cases, with larger values suggesting more accurate classification. Additionally, both LMR and BLRT were employed to compare the fit difference between class *k* and class *k* – 1. A significant result indicates that class *k* is superior to class *k* – 1.

We computed five LCA models, and the fit indices of the models were presented in Table 2. The number of models (1‐5 class models) was incrementally increased from one class until the number of classifications was not sufficiently justified (Hori et al., 2017). The AIC, BIC, and aBIC gradually decreased with an increase of the number of latent classes. The entropy values for the 1‐ to 5‐class models were all 0.93 and above, indicating a relatively high correct classification rate. If the choice of model only considered the information criteria and entropy, then both the 4‐class and 5‐class models fit better. However, we continued to examine BLRT and LMR in this study, owing to the LMR for the 5‐class model not indicating significance, indicating that the 4‐class model fit was better than the 5‐class fit. Therefore, the 4‐class model was finally selected as the best model.

**TABLE 2:** The indexes of model fit.

Latent class	Log likelihood	AIC	BIC	aBIC	Entropy	LMR (*p*)	BLRT(*p*)	Class probability
1‐Class	−120283.48	240966.95	242077.51	241442.11	‐	‐	‐	1
2‐Class	−113094.42	226990.83	229217.49	227943.51	0.94	<0.001	<0.001	0.40/0.60
3‐Class	−110504.10	222212.19	225554.95	223642.40	0.93	<0.01	<0.001	0.29/0.40/0.31
4‐Class	−108588.31	218782.61	223241.48	220690.35	0.94	<0.001	<0.001	0.22/0.36/0.25/0.17
5‐Class	−107290.92	216589.83	222164.80	218975.09	0.94	0.76	<0.001	0.20/0.22/0.11/0.21/0.26

Abbreviations: AIC = Akaike information criterion; BIC = Bayesian information criterion; aBIC = sample size‐adjusted BIC; LMR = Lo–Mendell–Rubin; BLRT = bootstrapped likelihood ratio test.

**FIGURE 1:**
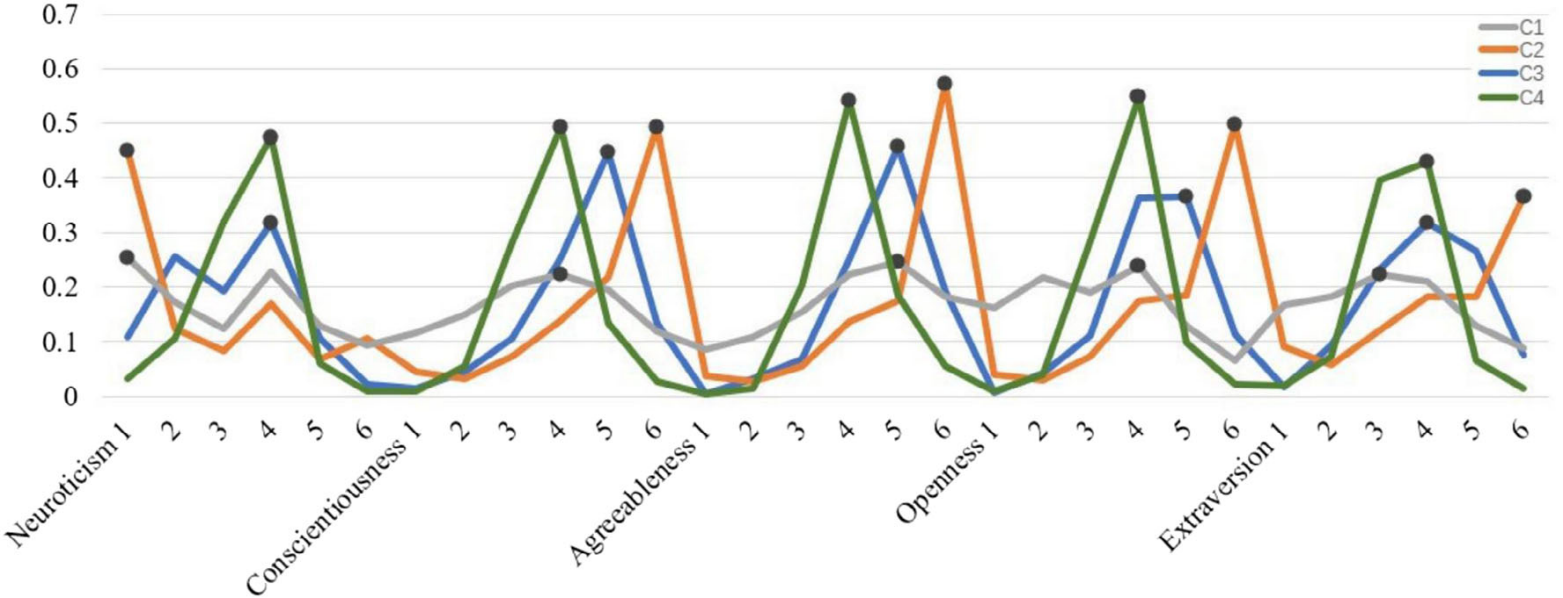
Profiles of average response probabilities for the Personality Scale. The values on the x‐axis refer to scale scores, and the y‐axis refers to probability (*n* = 1906). The black circle on each line represents the maximum tendency in the respective dimension.

The LCA divided participants into four classes by their patterns of response probabilities for personality. We calculated the average response probabilities for each endorsing item used to describe the features of each class (C1, C2, C3 and C4) in the model and further obtained the response tendencies of four personality classes across five dimensions (see Figure 1).

In terms of average response probabilities, C1 displayed high “very inconsistent” (score = 1) in the neuroticism dimension, “slightly consistent” (score = 4) in conscientiousness and openness, and a stronger inclination towards “moderately consistent” (score = 5) in agreeableness. However, C1 showed “slightly inconsistent” (score = 3) in the extroversion dimension. Additionally, the probability values of C1 were lower than of the other classes in all five dimensions, and the distribution of scores exhibited similar features to the overcontrolled class. So, we also named C1 as the *overcontrolled class*. It is worth noting that C1 accounted for 17% of the total participants. On the other hand, C2 demonstrated high “very inconsistent” in the neuroticism dimension, but high “very consistent” (score = 6) in all other personality dimensions, with probability values at a high level. As a result, we labeled C2 the *resilient class*, and it included 22% of all participants.

The personality types of C3 tended to be “slightly consistent” on the neuroticism and extroversion dimensions, and “moderately consistent” on the other three dimensions. Additionally, we found that the choice preference of C3 among the four personality dimensions (extroversion, agreeableness, conscientiousness, and openness) fell between C2 and C4. C3 was the largest class, comprising 36% of the total participants, and was therefore named the *moderate class*. As for the response probabilities of C4, it showed “slightly consistent” across all five dimensions of personality and had a high probability score, leading to its classification as the *vulnerable class*. It is worth noting that C4 accounted for 25% of the total participants.

A further examination of the differences among the big five personality traits within four classes of personality types (see Table 3) showed that the four classes were significantly different on the five personality traits using a one‐way ANOVA (*F*_Neuroticism_ = 59.49, *p* < .001, *η* ^2^ = 0.09; *F*_Openness_ = 519.86, *p* < .001, *η* ^2^ = 0.45; *F*_Conscientiousness_ = 345.67, *p* < .001, *η* ^2^ = 0.35; *F*_Extroversion_ = 231.11, *p* < .001, *η* ^2^ = 0.27; *F*_Agreeableness_ = 319.71, *p* < .001, *η* ^2^ = 0.34). The results were utilized to gain insights into the variations in mean scores among the four classes on the dimensions of personality traits, further confirming the characterization of the personality types.

**TABLE 3:** The differences on the big five personality traits between the four latent classes of personality.

Variable	Personality type (*M* ± *SD*)				*F*	Post hoc tests
	C1 (*n* = 319)	C2 (*n* = 427)	C3 (*n* = 692)	C4 (*n* = 468)		
Neuroticism	24.77 ± 8.90	20.84 ± 10.23	24.88 ± 6.67	27.59 ± 4.46	59.49***	C2 < C1 < C3 < C4
Openness	25.12 ± 6.86	39.43 ± 6.65	35.05 ± 4.63	29.96 ± 3.56	519.86***	C1 < C4 < C3 < C2
Conscientiousness	28.66 ± 7.73	39.42 ± 6.40	35.78 ± 4.40	30.12 ± 3.82	345.67***	C1 < C4 < C3 < C2
Extraversion	25.71 ± 6.30	35.23 ± 7.19	31.58 ± 4.88	28.00 ± 3.43	231.11***	C1 < C4 < C3 < C2
Agreeableness	31.82 ± 6.69	40.83 ± 5.62	37.65 ± 4.23	32.33 ± 3.87	319.71***	C1 < C4 < C3 < C2

*** *p* < .001.

The post hoc tests showed a significant difference in scores among the four classes in the openness, conscientiousness and extroversion dimensions (*p*s < .01), and exhibited C1 < C4 < C3 < C2. But when considering the neuroticism dimension, there was no significant difference observed between C1 and C3 (*p* > .05). However, the differences between pairs for all other classes were significant (*p*s < .001), and C2 scored the lowest, followed by C1 and C3, and the highest score was C4. Also, in the agreeableness dimension, there was a non‐significant difference between C1 and C4 (*p* > .05), whereas all other classes were significantly different from each other (*ps* < .001), with the lowest score for C1, followed by C4 and C3, and the highest score for C2.

### Comparison of nomophobia scores among the four classes of personality

Following the results of the LCA, we used a one‐way ANOVA to explore the relationship between the four personality types and nomophobia (see Table 4). The results showed that all four personality types were significantly different on the “total scores of nomophobia scale” and the “fear of being unable to obtain information” (*F*_total_ = 21.12, *p* < .001, *η* ^2^ = 0.03; *F*_information_ = 13.07, *p* < .001, *η* ^2^ = 0.02). Post hoc tests showed that C2 had the lowest scores for total score and the “fear of being unable to obtain information” dimension, followed by C1, C4, and C3, respectively. It was also found that there was a non‐significant difference between pairs of C1 and C2, and C4 and C3 (*p*s > .05). However, all other classes were significant differences in pairs of comparisons (*p*s < .01).

**TABLE 4:** Comparison of nomophobia scores among the four classes of personality using one‐way analysis of variance (ANOVA).

Variable	Personality type (*M* ± *SD*)				*F*	Post hoc tests
	C1 (*n* = 319)	C2 (*n* = 427)	C3 (*n* = 692)	C4 (*n* = 468)		
Nomophobia sum score	60.52 ± 21.65	59.84 ± 24.35	67.70 ± 19.32	67.49 ± 15.07	21.12***	C2 < C1 < C4 < C3
Information	14.82 ± 5.63	14.37 ± 6.30	16.09 ± 5.18	16.08 ± 3.98	13.07***	C2 < C1 < C4 < C3
Convenience	15.06 ± 6.70	13.82 ± 7.52	16.60 ± 5.96	16.89 ± 4.85	24.42***	C2 < C1 < C3 < C4
Contact	16.96 ± 7.01	18.57 ± 7.14	19.52 ± 5.59	18.53 ± 4.70	13.21***	C1 < C4 < C2 < C3
Internet connection	13.69 ± 6.77	13.09 ± 7.72	15.49 ± 6.17	15.99 ± 4.65	21.94***	C2 < C1 < C3 < C4

*Note*: Nomophobia sum score = total scores of “Nomophobia Scale”; Information = “fear of being unable to obtain information”; Convenience = “fear of losing convenience”; Contact = “fear of losing contact”; Internet connection = “fear of losing Internet connection.”.

*** *p* < .001.

For the “fear of losing convenience”, there were significant differences among the four classes (*F*_convenience_ = 24.42, *p* < .001, *η* ^2^ = 0.04). Post hoc tests showed that the “fear of losing convenience” scores were C2, C1, C3, and C4 in increasing order, but the differences between C3 and C4 were not significant (*p* > .05), while the pairs of other classes were significant differences (*p*s < .05).

For the “fear of losing contact”, there were significant differences among the four classes (*F*_contact_ = 13.21, *p* < .001, *η* ^2^ = 0.02). Post hoc tests showed that C1 had the lowest scores, followed by C4 and C2, and that C3 had the highest scores. However, there were non‐significant pairs of comparisons between C2 and C4, and C2 and C3 (*p*s > .05). All other classes had significant differences between pairs of comparisons (*p*s < .05).

Finally, for the “fear of losing Internet connection”, there was a significant difference in scores among the four classes (*F*_Internet connection_ = 21.94, *p* < .001, *η* ^2^ = 0.03). Post hoc tests of pairs of comparisons revealed that low to high scores were obtained in the order C2, C1, C3, and C4, but there were non‐significant pairs of comparisons for C2 and C1, and C3 and C4 (*p*s > .05), whereas pairs of comparisons were significant for all other classes (*ps* < .001).

In addition to the one‐way ANOVA, we also used the BCH method to verify the outcomes (see Table 5). Our analysis showed that the variance results for the “fear of losing contact” dimension were different from the results obtained from the one‐way ANOVA. Specifically, C2 had a marginally significant difference from C3 (*p* < 0.05), and C2 scored lower than C3 in this dimension. However, all other variance results were consistent with the one‐way ANOVA results.

**TABLE 5:** Comparison of nomophobia scores among the four classes of personality using BCH.

Variable	Type	Between‐group variation				Overall chi‐square test
		C1	C2	C3	C4	
Nomophobia sum score	C1	0				57.06***
	C2	0.14	0			
	C3	25.85***	31.93***	0		
	C4	24.48***	31.21***	0.11	0	
Information	C1	0				35.02***
	C2	1.01	0			
	C3	11.86**	22.44***	0		
	C4	11.63**	22.96***	0.03	0	
Convenience	C1	0				64.35***
	C2	5.47*	0			
	C3	12.21***	41.73***	0		
	C4	17.11***	51.62***	0.58	0	
Contact	C1	0				35.22***
	C2	9.57**	0			
	C3	33.09***	5.72*	0		
	C4	12.21***	0.01	10.77**	0	
Internet connection	C1	0				63.03***
	C2	1.19	0			
	C3	16.46***	29.32***	0		
	C4	27.63***	45.56***	2.10	0	

*Note*: Nomophobia sum score = total scores of “Nomophobia Scale”; Information = “fear of being unable to obtain information”; Convenience = “fear of losing convenience”; Contact = “fear of losing contact”; Internet connection = “fear of losing Internet connection.”.

* *p* < .05.

** *p* < .01.

*** *p* < .001.

## DISCUSSION

In continuation of previous research on the higher prevalence of nomophobia among young individuals, this study focused on Chinese university students as participants. The objective was to explore the latent classes of personality traits and establish the relationship between these distinct personality types and nomophobia.

From the results of the LCA, university students' personality traits were categorized into four homogeneous groups. These personality types were referred to as the *overcontrolled class* (C1), *resilient class* (C2), *moderate class* (C3), and *vulnerable class* (C4). C1 exhibited low levels in all five dimensions of personality, with a notable preference for “slightly inconsistent” in the extroversion dimension. However, the choice preference for neuroticism leaned towards “very inconsistent”, while the mean score remained at a moderate level. Analyzing the researchers' summary of personality types, it became evident that C1 displayed traits aligning more with the overcontrolled type (Rosenström & Jokela, 2017). This type is characterized by sensitivity, introversion, self‐restraint, and limited socialization (Bohane et al., 2017; Donnellan & Robins, 2010). C2 demonstrated a preference for the “very inconsistent” option on the neuroticism dimension, and also had the lowest scores on this dimension. In the other four dimensions, the choice preferences for C2 all tended to be “very consistent” and had the highest mean scores. Regarding the general factor of personality (GFP), also known as the “Big One” personality,  it represents the highest‐order structure within the Big Five personality framework (Musek, 2007). C2 in this study were consistent with the distribution of “Big One” personality, which exhibited low levels of neuroticism and high levels of extroversion, conscientiousness, agreeableness, and openness. Researchers have identified that the “Big One” personality was associated with more enthusiasm for life, greater adaptability to change in the environment, and superior evolutionary quality (Dunkel et al., 2021; Fisher & Robie, 2019; Tackett et al., 2015).

C3 and C2 had similar choice preferences and mean scores, except in the neuroticism dimension. C3 fell between C2 and C4, exhibiting moderate levels of extroversion, agreeableness, conscientiousness, and openness. In terms of neuroticism, C3 showed a preference for “slightly consistent” and scored moderately, without any significant difference from C1. De Clercq et al. (2012) discovered that the moderate class fell between the various personality types and demonstrated moderate scores on all dimensional averages. It also had the highest proportion of individuals. As a result, we also designated it as the moderate class. While the personality traits of the *moderate class* were similar to *resilient class*, they were not as adaptive. Similarly, C4 was found to be “slightly consistent” on all five dimensions. The mean scores indicated that C4 had the highest level of neuroticism, while the other personality scores were relatively lower than other personality types. Because the personality distribution features of C4 showed high levels of neuroticism and low levels of extroversion, conscientiousness, agreeableness, and openness, C4 represented the vulnerable personality (De Clercq et al., 2012). Van Den Akker et al. (2013) pointed out that a vulnerable personality could have maladjustment problems for the environment. To summarize, this method is an innovative approach to the traditional analysis of personality. Instead of being divided into five dimensions, the big five personality traits are presented as a unified whole, reflecting an individual's personality characteristics. The description of personality traits is thus more complex, and highlights the interplay between personality traits and the diversity that exists within them.

From the results of the one‐way ANOVA and BCH, significant differences were found on the total scores and dimensions of nomophobia among the four classes. First, the post hoc tests for C1 and C2, as well as for C3 and C4, were similar for the variables “total scores of nomophobia scale”, “fear of being unable to obtain information”, “fear of losing convenience”, and “fear of losing Internet connection”. In particular, there was no difference between pairs of comparisons in “total scores of nomophobia scale”, “fear of being unable to obtain information”, and “fear of losing Internet connection”. The results indicated a higher prevalence of nomophobia in moderate and vulnerable individuals compared with overcontrolled and resilient individuals. This finding was related to the personality types analyzed above in this study. The recent advancement in mobile phone technology has enabled people to transfer information and solve problems, thereby bringing convenience to their daily lives through the Internet. Barańczuk (2019) showed that individuals who were vulnerable may adopt avoidance and emotion suppression, and therefore struggle to adapt to changes in their environment. Similarly, individuals of a moderate type also showed a lower level of adaptability and a higher preference for entertainment, aligning with the characteristics commonly observed in contemporary youth (Udayar et al., 2020). They relied on their phones more for regular entertainment and faced difficulties in adapting swiftly to being without their phones. On the other hand, resilient individuals possessed higher self‐regulation and social adaptation to reduce problem behaviors (Chen et al., 2022). Overcontrolled individuals were more self‐regulated and introverted, which made them less inclined to use and depend on mobile phones. This explained why individuals with vulnerable and moderate personalities tended to score higher than those with resilient and overcontrolled personalities in term of “total scores of nomophobia scale”, “fear of being unable to obtain information”, “fear of losing convenience”, and “fear of losing Internet connection” in this paper.

Second, C1 scored lower in the “fear of losing contact” variable compared with C4 and C3. C1 exhibited lower self‐expression owing to lower scores on dimensions such as extroversion and openness. Montag and Panksepp (2017) noted that individuals with low self‐expression tended to use mobile phones less frequently for communication and have lower levels of nomophobia. Previous research has also found that vulnerable individuals were more likely to seek social support from family and friends to cope with negativity. The absence of mobile phones represented a loss of contact for them (Alsubaie et al., 2019). Consequently, C4 exhibited a greater fear of losing mobile phone contact. Meanwhile, C3 was distinguished from C4 by conscientiousness, agreeableness, and openness. The correlation results of this study were consistent with previous studies, which revealed a significant positive correlation between three personality dimensions and the fear of losing contact (Chen et al., 2022; Horwood & Anglim, 2021; Liu et al., 2020). The personality traits of conscientiousness, agreeableness, and openness were all characterized by effective communication and interaction with others. Consequently, individuals who were moderate tend to be more concerned about losing contact.

Finally, the findings revealed significant differences between C1 and C2 in terms of the “fear of losing convenience” and “fear of losing contact”. However, the results of these differences were unexpected. Based on previous research of single personality traits, C2 had a lower score than C1 on nomophobia from the neuroticism perspective. But C1 had a lower score than C2 on nomophobia from the extroversion perspective. This paradoxical result further supports the main idea of our study, which emphasizes the importance of considering the five dimensions of personality from a holistic perspective. Future research could focus on investigating which specific personality traits play a central role in the “fear of losing convenience” and “fear of losing contact”.

### Implications

The research in this paper delved into the connection between personality and nomophobia, departing from the traditional variable‐centered approach to uncover significant findings on the correlation between personality traits and nomophobia. Initially, the study discussed the person‐centered personality theory introduced by Block and Block (1980), which includes ego‐control and ego‐resilience. Subsequently, various researchers have expanded on this theory by identifying additional personality types using different assessment tools. This paper, based on a large sample of Chinese college students, identified four distinct personality types through LCA, thereby enhancing person‐centered personality theory. Furthermore, the study has presented compelling evidence supporting the I‐PACE model (Brand et al., 2016), indicating that individual core traits influence different patterns of mobile phone usage, ultimately impacting the manifestation of nomophobia. Additionally, the study employed both a one‐way ANOVA and the BCH method to examine the relationship between personality types and nomophobia, addressing the limitations of using ANOVA in isolation and providing a more comprehensive analysis. The research underscores the prevalence of nomophobia among university students and advocates for proactive measures to mitigate nomophobia and phone addiction. It emphasizes the importance of considering individual factors in mobile phone usage, especially targeting the moderate and vulnerable groups, to promote a healthier lifestyle and academic success among students.

### Limitations of research

The findings of this study contribute to an understanding of the relationship between personality traits and nomophobia. However, there are some limitations that should be acknowledged. First, the scope of this paper was limited to college students, as nomophobia may manifest differently at different ages. It is therefore important to investigate whether personality types vary across different age groups. Second, the study was conducted with a Chinese sample, and it is uncertain whether the results can be generalized to Western populations. Finally, our study utilized a cross‐sectional design, which limits our ability to establish causality as it only captures a single point in time. Therefore, we cannot fully determine the causal relationship.

## CONCLUSIONS

The present study aimed to examine the combined impact of the five dimensions of personality on nomophobia. To achieve this, the study utilized LCA to categorize personality traits into four distinct types: overcontrolled, resilient, moderate, and vulnerable. Subsequently, a one‐way ANOVA and BCH were conducted to assess the association between these personality types and nomophobia. The results indicated that college students in the moderate and vulnerable groups were more susceptible to developing nomophobia, whereas those in the overcontrolled and resilient groups exhibited better adaptation. It is important to note that the participants in this study were Chinese college students, which may limit the generalizability of the findings. Future research should consider expanding the age range of the sample population.

## CONFLICT OF INTEREST STATEMENT

The authors report no conflicts of interest in this work.

## ETHICS STATEMENT

This research was conducted according to the Declaration of Helsinki and was approved by the Ethics Committee of Tianjin Normal University (XL2020‐08). Each participant provided informed consent.

## Data Availability

The data are not publicly available as the information contained in the data may compromise the privacy of the participants. The data can be obtained from the corresponding author.

## Footnotes

1
*Note*: variable 6, Nomophobia = total scores of the Nomophobia Scale, and variables 7–10 are four subscales from the Nomophobia Scale; Information = “fear of being unable to obtain information”; Convenience = “fear of losing convenience”; Contact = “fear of losing contact”; Internet Connection = “fear of losing Internet connection.” The values in red font indicate numbers of 0.059 (excluding the positive or negative sign), but the significance differs due to rounding of the *p*‐values.

**
*p* < 0.01.

*
*p* < 0.05.
